# Exome/Genome Sequencing in Undiagnosed Syndromes

**DOI:** 10.1146/annurev-med-042921-110721

**Published:** 2023-01-27

**Authors:** Jennifer A. Sullivan, Kelly Schoch, Rebecca C. Spillmann, Vandana Shashi

**Affiliations:** Division of Medical Genetics, Department of Pediatrics, Duke University School of Medicine, Durham, North Carolina, USA

**Keywords:** exome sequencing, genome sequencing, next-generation sequencing, undiagnosed diseases, rare/ultrarare diseases, Mendelian diseases

## Abstract

Exome sequencing (ES) and genome sequencing (GS) have radically transformed the diagnostic approach to undiagnosed rare/ultrarare Mendelian diseases. Next-generation sequencing (NGS), the technology integral for ES, GS, and most large (100+) gene panels, has enabled previously unimaginable diagnoses, changes in medical management, new treatments, and accurate reproductive risk assessments for patients, as well as new disease gene discoveries. Yet, challenges remain, as most individuals remain undiagnosed with current NGS. Improved NGS technology has resulted in long-read sequencing, which may resolve diagnoses in some patients who do not obtain a diagnosis with current short-read ES and GS, but its effectiveness is unclear, and it is expensive. Other challenges that persist include the resolution of variants of uncertain significance, the urgent need for patients with ultrarare disorders to have access to therapeutics, the need for equity in patient access to NGS-based testing, and the study of ethical concerns. However, the outlook for undiagnosed disease resolution is bright, due to continual advancements in the field.

## INTRODUCTION

Undiagnosed diseases are defined as disorders that are refractory to standard clinical and laboratory evaluations and encompass a variety of rare/ultrarare disorders, the majority (~80%) of which are genetic in etiology (Mendelian) (1). Individually, they are uncommon; a rare disease is defined as one that affects <200,000 individuals and an ultrarare disease affects <2,000 individuals in the United States. However, collectively, there are about 7,000 of these disorders affecting 6–8% of the US population, thereby causing a significant public health and societal/economic impact, in addition to the personal medical, financial, and psychosocial toll on the patients (2, 3). Until a little over a decade ago, these rare/ultrarare disorders could only be diagnosed by sequential targeted genetic testing, which yielded a diagnosis in ~50% of cases (4). The patients who remained undiagnosed experienced a prolonged diagnostic odyssey with few options beyond further Sanger gene sequencing, which allows only a single strand of DNA to be sequenced at a time (2, 4–9).

Exome sequencing (ES) and genome sequencing (GS), performed using a next-generation sequencing (NGS) platform, have transformed the approach to these disorders. ES involves the simultaneous sequencing of millions of short fragments of the coding region, whereas in GS, both the coding and noncoding regions are sequenced. ES/GS has yielded previously unimaginable diagnoses and new disease gene discoveries in about 25–50% of undiagnosed patients (1, 10–19). Thus, NGS has enabled precision medicine for patients with undiagnosed rare/ultrarare diseases, with the genomic information being utilized for diagnosis, prevention of complications, and customized medical management of the individual patients (20–25). Currently, ES is widely implemented in medical genetics practice (23, 26). Due to its enhanced ability to detect copy number/noncoding variants, GS has a 10–15% higher diagnostic yield than ES (27–29) and is likely to be implemented widely as costs drop. In the next sections, we describe the history and process of ES and GS, their application to undiagnosed diseases, the underlying challenges, and future directions.

### History of Next-Generation Sequencing

**Figure 1:**
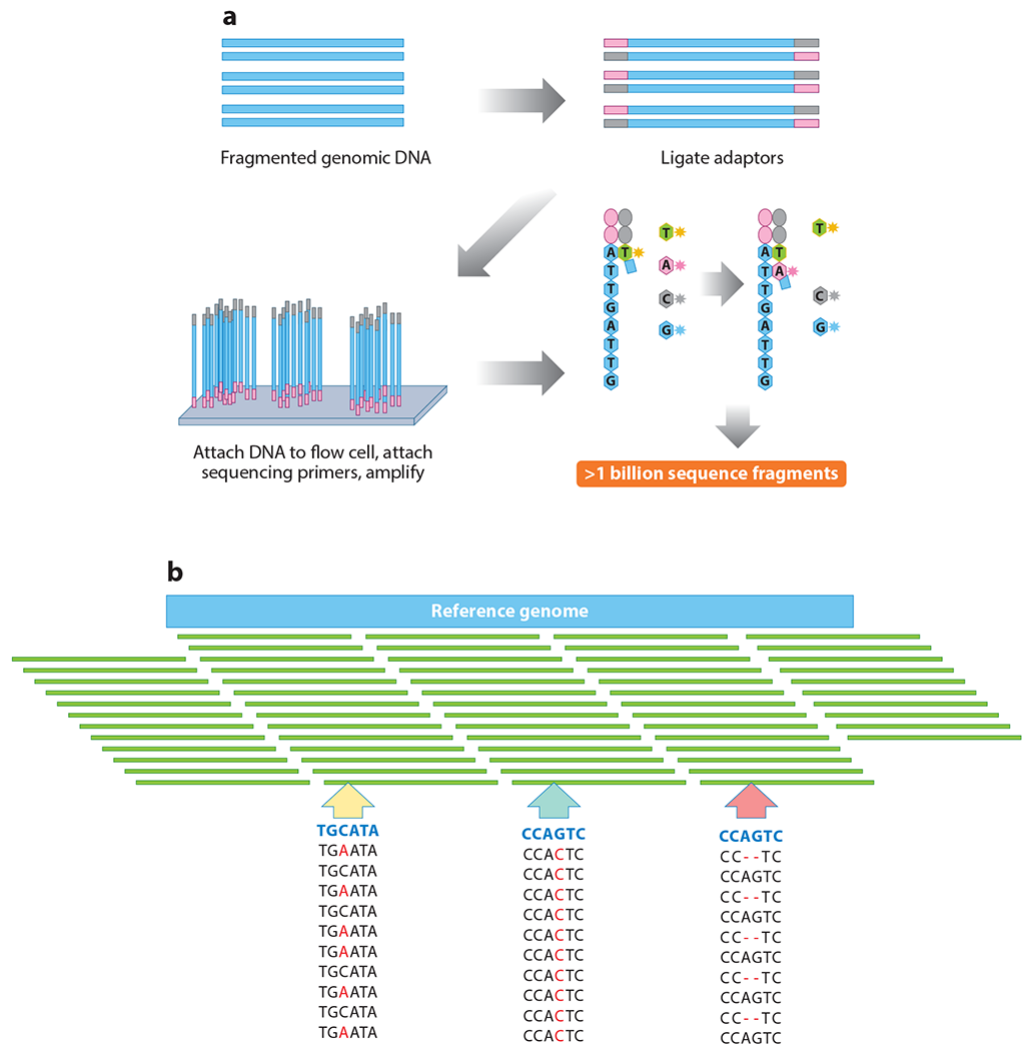
Next-generation sequencing with Illumina technology. (*a*) Library preparation and sequencing. DNA is fragmented and adapters ligated to create a sequencing library. Samples are then reassembled in the genetic sequence used for data analysis. (*b*) Alignment and variant calling. Short-read DNA fragments are aligned to the reference genome for variant calling. Examples of variants of interest include findings such as identification of a heterozygous missense variant (*yellow arrow*), a homozygous missense variant (*blue-green arrow*), and/or a two-base-pair heterozygous deletion (*pink arrow*).

The first human genome sequence was completed in 2003 by means of traditional Sanger sequencing, at a cost estimated at $3 billion, with international collaborations and governmental funding of the Human Genome Project (30, 31). This was the harbinger of the NGS revolution. In the subsequent decade, with National Institutes of Health (NIH) and private investment, the development of high-throughput sequencing machines enabled the implementation of NGS. The first NGS platform was introduced in 2000 by 454 Life Sciences (32), followed by rapid advances in the technology such that the commercial company Illumina was able to facilitate laboratories offering clinical ES by the end of the decade. Unlike traditional Sanger single-gene sequencing, wherein a single strand of DNA of a gene is amplified, the Illumina technology consists of massively parallel short-read sequencing, in which millions to over one billion DNA fragments of approximately 100–500 base pairs are simultaneously generated across the exome or the genome for alignment and analysis (see Figure 1).

As NGS technologies were evolving, there was simultaneous rapid application of these to rare/ultrarare undiagnosed diseases, starting around 2008. The first effort involved applying ES to four unrelated individuals with a genetic disorder, Miller syndrome, in 2010, to identify the underlying gene (33). Soon after, another study applied trio ES, by testing the child and both biological parents, to unrelated probands with intellectual disability and found that de novo variants were the most common cause (34). Interestingly, this early discovery has been confirmed with subsequent studies; in countries where consanguinity rates are low, approximately two-thirds of severe rare/ultrarare neurodevelopmental disorders are attributed to de novo damaging variants that reduce reproductive fitness (27, 35). Early studies of ES utility demonstrated a diagnostic rate of 18–50% in cases studied, and within the next few years, ES became standard clinical practice. GS is now emerging as a clinical tool; thus, this model of “genomic medicine” utilizing NGS is standard in medical genetics practice when undiagnosed patients are evaluated (7, 13–15, 17, 36).

### Next-Generation Sequencing Process

The NGS workflow involves several sequential steps, which have been previously described (37). An important distinction is that ES captures only the coding regions of the genes (1–2% of the genome), whereas GS also captures the noncoding regions. For clinical NGS, the prioritized variants are interpreted and classified into five categories, according to guidelines provided by the American College of Medical Genetics and Genomics (ACMG) in 2015. Laboratories performing NGS most often only report variants interpreted in three of the ACMG categories: “pathogenic,” “likely pathogenic,” or “variants of uncertain significance” (VUS) (38). Clinicians then interpret the reported variants in the clinical context to make the final determination of whether a diagnosis has been made; this may require reconsideration of the phenotyping or further downstream testing, if the phenotypic concordance is not immediately obvious (39).

## BEST PRACTICES FOR ORDERING EXOME AND GENOME SEQUENCING

Multiple clinical reports and research studies have emphasized the value of ES and more recently GS in patients with undiagnosed rare/ultrarare syndromes that have been refractory to traditional diagnostic approaches. Numbers enrolled in clinical studies have ranged from a handful of patients to thousands, including data from commercial laboratories that perform ES/GS, dedicated “exome clinics” in academic institutions, and standard medical genetics clinics (13, 14, 17, 19, 40–42). Large-scale research networks have also contributed data on the best practices and utility of ES and GS in undiagnosed rare/ultrarare diseases. The diagnosis rates with ES/GS vary from 25% to 50%, with the higher end being associated with GS (14, 15, 17, 19, 29, 43). De novo variants are highly prevalent in rare/ultrarare diseases, especially in severe neurodevelopmental disorders, wherein they account for two-thirds of causal variants. This has changed the landscape of reproductive risk assessment for these devastating disorders (34, 44).

### Indications for Exome and Genome Sequencing

Individuals with clinical presentations highly suggestive of a specific disorder should have targeted testing for that condition first. Examples include individuals with features of a chromosomal disorder such as Down syndrome, a known family history of a disorder, or specific clinical features of a disorder not detectable by sequencing (such as fragile X syndrome). Based on a review of the existing literature, the ACMG strongly recommends that ES/GS be used as a first-line (or second-line, if indicated) test in patients with one or more congenital anomalies, developmental delay, and/or intellectual disability with onset prior to age 18 years, citing clinical utility with limited evidence for negative outcomes (45). Higher diagnostic yield is achieved when ES/GS is applied to individuals with disorders involving hearing, vision, the musculoskeletal system, the skeletal system, multiple congenital anomalies, skin, the central nervous system, and the cardiovascular system (41).

### Singleton Versus Trio Analysis

The family structure used for testing is crucial to maximize the yield of ES and GS. Classical trio (child and both biologic parents) sequencing can increase yield by 10–15% due to the detection of de novo variants and compound heterozygous variants (27).

### Ordering Exome Sequencing Versus Genome Sequencing

ES is effective in detecting most variants associated with rare/ultrarare Mendelian diseases, since coding variants account for 85% of disease-associated variants for Mendelian disorders. GS can detect more types of variants than ES, especially copy number variants (CNVs) (indels larger than 50 base pairs), as well as noncoding variants (e.g., splice variants) and trinucleotide repeat variants. GS also provides slightly more uniform coverage of the coding regions than ES (reviewed in 46). However, GS is more computationally intensive and generates more data, which require more storage capacity. In the setting of a prior nondiagnostic ES, GS can provide an additional 10–15% of diagnoses (27, 28). However, many of these incremental diagnoses on GS are actually due to the detection of coding variants (e.g., indels, CNVs, and single-nucleotide variants) rather than noncoding variants, since GS allows for better detection of indels/CNVs and more uniform coverage of the exome, permitting better detection of single-nucleotide variants (27, 47).

### Value of Periodic Reanalysis

With 40–75% of patients not obtaining a diagnosis with ES/GS, an avenue to achieve more diagnoses is to reanalyze the NGS data after a time lapse of a year or more (27, 48). Due to rapid accumulation of new data, VUS can be reclassified and new variants can be identified with a time-lapsed reanalysis of NGS data, even with the same pipeline. Clinical studies have reported a resolution rate of 10–15% with such reanalysis, mostly due to interim new gene–disease associations and variant pathogenicity updates. Studies report 25–50% higher resolution rates due to more specific efforts such as realigning the genomic data to new reference genomes and using updated bioinformatics tools (49–51). Commercial laboratories offer a reanalysis at least a year after the ES/GS was performed, most often at no cost, due to the value of the reanalysis. Clinicians must reconcile variants on reanalysis with the patient’s phenotype to confirm or refute a diagnosis. Finding diagnostic resolution with reanalysis provides cost efficiency for patients who do not initially obtain a diagnosis with ES/GS.

### Obtaining Informed Consent, Determining Preferences for Secondary/Incidental Findings, and Discussing Possible Risks

Individuals or families should be informed of the potential benefits and risks as well as limitations of undergoing ES/GS, and written informed consent should be obtained before the test is initiated. Starting in 2013, with periodic updates since, the ACMG has identified a gene list that clinical laboratories will proactively evaluate in all patients who undergo ES/GS and consent to receive this information (52). Genes are listed if a medical intervention is indicated for the associated disorder, such that a diagnosis would lead to preventive surveillance or specific treatments (e.g., *BRCA1* and *BRCA2* genes associated with hereditary breast cancer). This secondary gene list currently stands at 78 (from an initial 59) and will be updated regularly as the data evolve (53). Incidental findings are defined as variants within genes that are not included in the ACMG secondary gene list yet are medically actionable, and laboratories vary considerably in the type and number of genes in which they report incidental findings. Secondary/incidental findings are reported in 2–3% of individuals who undergo ES/GS (17). Numerous studies have found that both patients and providers view secondary/incidental findings as being important to optimizing care, with >90% patients consenting to be informed of these findings (54). This practice of reporting secondary/incidental findings is highly prevalent in clinical practice across different countries but less likely to be adopted in research studies.

In addition to the preferences for learning about secondary/incidental findings, pretest counseling should include discussion of the expected outcomes of testing and potential risks, such as insurance discrimination and implications for extended family members (55). Misattributed familial relationships may also be revealed during the process of ES/GS, and families should be informed of this possibility before proceeding with the testing (56).

## LIMITATIONS IN THE APPLICATION OF EXOME AND GENOME SEQUENCING FOR UNDIAGNOSED DISEASES

Despite the unprecedented advances in the diagnoses and new gene associations for Mendelian diseases directly attributable to ES/GS, the fact remains that most individuals who undergo ES/GS due to undiagnosed diseases do not obtain a diagnosis. The reasons for this vary and depend on the type of sequencing (ES versus GS), family structure (trios versus singletons), and individual analytical differences between the laboratories. For example, commercial labs may not prioritize and report variants that are discordant with the phenotype and thus phenotype expansions may be overlooked; in one study, 22% of variants that were prioritized as causal had not been reported by commercial laboratories due to phenotypic mismatch (28). However, beyond all sequencing and analytical differences, it is evident that ES/GS does not provide diagnoses in >50% of individuals who are sequenced. This section describes four reasons: VUS, the existence of phenocopies of disorders, limitations of the technology, and discordant interpretations.

Defined as variants that lack evidence for pathogenicity or benignity, VUS are the most frequent type of variant reported with NGS, with 40–70% of clinical reports as well as research studies reporting them (39, 57). Most VUS are coding missense variants, and the difficulty in accurately predicting their impact, as well as other synonymous variants (e.g., in-frame deletions) and noncoding variants, poses a major limitation to achieving diagnoses and new gene–disease associations (12, 58). Time itself (due to continual accrual of data on variants and genes) can provide clarity for 10–15% of VUS (27). However, most VUS remain unresolved, posing a clinical and research conundrum. Case-matching, functional assays such as RNA-Seq, and animal modeling of variants/genes are options to pursue VUS further but require considerable time and resources that are scarce in a clinical setting.

Phenocopies of Mendelian disorders can pose diagnostic challenges. Many patients who do not obtain a diagnosis with ES/GS may have presenting clinical features that appear to be Mendelian but may be due to other causes such as complex multifactorial etiologies (e.g., congenital anomalies such as cleft lip, or anomalies and developmental delays due to teratogen exposures).

NGS is subject to technological limitations on the types of variants that can be detected. For example, polymerase chain reaction (PCR) amplification causes GC-rich areas of the exome to not be amplified correctly in ES. PCR-free GS can overcome this limitation. Furthermore, due to the short-read nature of current clinical NGS, areas of the genome where there are repeated DNA sequences are difficult to align and obtain an accurate sequence for. Although GS detects CNVs and structural variants, due to the nature of the short reads, some of these can be missed. Epigenetic abnormalities are not detected by NGS, and thus these disorders, which can appear to be Mendelian, can be missed. We discuss in the next section the potential for long-read sequencing to overcome these challenges.

Discordance in variant interpretation is another reason NGS may not lead to a diagnosis. Although ACMG guidelines provide an evidence-based framework for variant interpretation, considerable differences exist between laboratories that offer clinical ES/GS, resulting in a discordant interpretation rate varying from 11% to 26% (59). It is beyond the scope of this article to describe in detail all the reasons for these discrepancies, but common causes include the different computational tools used to predict the consequences of a variant, differences in variant threshold frequency cutoffs (for both population data and disease-associated data), and not considering differences in transcript isoforms (60). These differences in variant interpretation can be resolved with more data sharing through public resources such as ClinVar (61) (wherein variants associated with diseases are catalogued, with the associated evidence and phenotypic information), and curation of genes and their variants to determine their clinical relevance, as is occurring through ClinGen (62). Another important discordance occurs when the clinician disagrees with the variant interpretation of clinical laboratories, causing a conundrum in making diagnoses. This can occur in ~10% of cases and requires significant patient and clinician time and resources, such as downstream additional phenotyping, to resolve (39). The importance of phenotypic consideration in variant interpretation by clinicians cannot be overemphasized, since it can prevent both misdiagnoses and missed diagnoses (60).

## IMPACT OF EXOME AND GENOME SEQUENCING ON UNDIAGNOSED SYNDROMES

### Discovery of Novel Disease Genes and Disorders

There has been an exponential increase in the detection of novel disease genes, due to ES and GS. Indeed, there have been more than 250 new disease gene discoveries annually for the last few years, compared to a handful in the decades prior to 2010, speaking to the power of ES/GS in unraveling the genetic underpinnings of Mendelian diseases (1, 11). As a result, currently over 4,600 genes are associated with Mendelian disorders, with gene discovery continuing steadily, and it is estimated that ES/GS may detect as many as 10,000 other genes to be associated with Mendelian conditions (12). Since gene discovery for rare diseases is likely to have been completed with the cumulative gene discoveries of the last few decades, novel candidate genes detected by ES/GS are now increasingly associated with ultrarare disorders, often in an *n* = 1 setting. This context can pose a challenge in establishing the gene’s association with disease. However, international case-matching efforts such as GeneMatcher and Matchmaker Exchange have resulted in rapid identification of cohorts of patients across the globe large enough to surmount the *n* = 1 conundrum and enable confirmation of gene–disease associations (63, 64). This is especially valuable for de novo variants that are not easily amenable to traditional positional cloning approaches, which require multiple affected individuals across families (65). Animal modeling of gene variants and RNA-Seq can also provide functional validation for candidate genes, to help establish a disease association (27).

### Insights into the Pathogenesis of Rare and Ultrarare Disorders

The cumulative impact of NGS technology, ever-expanding public databases, and increased international connectivity has resulted in a refined ability to not only identify but also deeply explore overlapping and distinct phenotypes observed in patients with variants that have compelling bioinformatics signatures. In practice, this ability has led to a wide range of insights into the pathogenesis of genes. ES/GS technology has resulted in not only the previously mentioned exponential rate of novel gene discovery but also novel insights into the interplay between disease pathogenesis and phenotypic expression. In recent years, as shown in Table 1, ES/GS findings have linked novel phenotypes with established genes (66), and further delineated unique, and often strikingly different, phenotypes associated with individual variants (67), specific gene regions (68), types of genetic variant (69), and different molecular consequences (loss of function versus gain of function) (70).

### Changes to Medical Management

Medical management changes occur in 25–30% of patients who obtain a diagnosis with ES/GS. Such changes include specific treatments, surveillance for additional manifestations, eligibility for clinical trials, and accurate reproductive risk assessment (71, 72). Importantly, the broad scope of ES/GS can, in rare instances, dramatically expedite diagnosis-specific interventions for patients who present with a nonspecific phenotype (73). Further, the rapid identification of specific variants fostered innovative interventions such as the repurposing of existing medications to target the biochemical pathway impacted by pathogenic variants (74, 75), and the development of antisense oligonucleotides targeted to specific pathogenic variants that inhibit aberrant protein production (76). With better refinement of NGS technologies, these tangible benefits to patients are likely to increase.

#### Cost efficiency of exome and genome sequencing in undiagnosed disease patients.

As a prelude to any discussion of cost efficiency in rare/ultrarare diseases, it is important to emphasize that traditional cost efficiency models/expectations are difficult to apply to undiagnosed diseases. When new treatments do occur due to diagnoses, they can be more expensive (e.g., enzyme replacement therapy for a lysosomal disorder). Changes in medical management can increase costs after diagnoses are achieved (e.g., periodic medical screening surveillance to detect new manifestations over time). Furthermore, for most rare/ultrarare disorders, no changes in medical management result from diagnoses, so costs for management of the symptoms can be unchanged, although costs related to further diagnostic testing (genetic and nongenetic) would be expected to decrease after a diagnosis is made.

A number of studies have explored the cost efficiency of ES/GS. ES cost efficiency studies have reported that early application of ES can result in cost savings in diagnostic tests or at least can be cost neutral compared to traditional genetic testing (77, 78). Attesting to the difficulty in using traditional cost efficiency models for rare/ultrarare disorders, one study found that the downstream medical costs after GS were dependent on the symptoms of the patients, rather than upon a diagnosis; however, further diagnostic testing costs dropped after GS (79). A comprehensive review of 36 published studies on cost efficiency of ES/GS reported that there was insufficient evidence to prove their cost efficiency, but this was likely due to small sample sizes and unclear/varying methodology in many studies (80). Recent emerging data are attesting to the value and veracity of GS over standard diagnostics, with cost neutrality at the very least (81). A recent meta-analysis reported that GS could be cost effective in the diagnostic workup of undiagnosed infants and children but that further economic evaluations are needed for comparing GS versus ES (82). Nonetheless, with the cost of ES being lower (~$1,000) than many gene panels and with the cost of GS expected to decrease with time, and given the ability of ES/GS to make diagnoses that would otherwise be difficult to achieve, we believe that ES and GS are here to stay. In the future, GS will likely be implemented more often than ES in the diagnostic approach to patients with mystery symptoms.

#### Patients’ perspectives on next-generation sequencing.

Being undiagnosed and experiencing an odyssey in search of a diagnosis can result in considerable psychosocial distress for patients and their families, with over one-third reporting anxiety and depression and most experiencing chaos in their lives (83, 84). When ES/GS results in diagnosis of an ultrarare disorder, with only a handful of patients similarly affected, there is little information on prognosis, and potential specific treatments are intangible, leaving patients and families to feel that they only “have a gene” with little clarity as to the next steps (85). The reality is that most patients diagnosed with ultrarare disorders have no specific treatments available, which results in variable, and sometimes low, perceived utility of ES/GS for some families (86). This is an important avenue to explore with focused research but requires commitment from funding agencies and the pharmaceutical industry; due to poor market potential, pharmaceutical companies’ commitment to drug development for ultrarare disorders is seriously lacking.

## FUTURE DIRECTIONS

NGS is here to stay, but it is unclear at this point whether current short-read ES/GS will be surmounted by newer NGS technologies such as long-read sequencing. Long-read sequencing provides the following advantages (87):

1. PCR-free sequencing captures the areas of the genome that are recalcitrant to short-read ES/GS.
2. Better ascertainment of larger structural rearrangements/CNVs, due to longer DNA fragments (several kilobases to 1 Mb, as opposed to 300 base pairs with short-read NGS).
3. Detection of transcript isoforms and epigenetic variations.

However, the sensitivity with which single-nucleotide variants can be detected has been a concern with long-read sequencing due to error rates of over 10%. Commercial entities that offer long-read sequencing have made sequencing chemistry adaptations to overcome this limitation. Two well-known commercial entities that offer long-read sequencing are PacBio (single-molecule realtime DNA sequencing) and Oxford Nanopore Technologies (sensors detect the DNA molecules due to ionic current changes) (88, 89). Remarkably, both methods were recently utilized to complete gaps (~8%) in the human genome, >20 years after the completion of the Human Genome Project (90). Currently, long-read GS is not available clinically and is prohibitively expensive for research purposes; there are few reports of long-read sequencing being applied to undiagnosed rare/ultrarare diseases (57, 91). With research consortia applying long-read sequencing to these disorders on a larger scale, such as the NIH GREGoR Consortium, there will be more clarity on the utility of these technologies for undiagnosed diseases. More tractable approaches to resolving VUS, with better population and disease data and scalable functional assays, are crucial to decrease the uncertainties these variants pose to patients and providers (58). Multi-omics will complement NGS and enhance new disease discovery and diagnosis by utilizing technologies beyond DNA sequencing, such as RNA-Seq (transciptomics), metabolomics, epigenomics, and proteomics (92).

The rapid expansion of clinical ES and GS requires more consideration of the impact of these technologies on patients and their families, particularly on “next steps” for families that get a diagnosis of an ultrarare disorder, such that there can be a path to better therapeutics. While gene therapy and oligonucleotide treatments can be patient specific, they are inherently not scalable, and thus other more far-reaching options such as drug repurposing need to be pursued for these devastating disorders. Patient–scientist partnerships are likely to drive the field much further than scientists alone, with social media being an important vehicle to connect patients to one another and to the scientific community, in addition to the more traditional disorder-specific foundations (93, 94). More research is necessary to assess the advantages of patient–scientist partnerships, as well as disadvantages such as privacy concerns, misinformation, and misinterpretation. Ethical concerns related to ES/GS of newborns and fetuses to diagnose rare/ultrarare disorders prior to the onset of symptoms have not been discussed in this article but need further scrutiny (95, 96). Finally, we need to increase participation in ES and GS by patients who are medically underserved, such as patients in rural areas and racial and ethnic minorities, who participate in genomic medicine at much lower rates (97).

## CONCLUSION

NGS technologies have revolutionized the diagnostic approach to rare/ultrarare Mendelian disorders, allowing for previously impossible diagnoses, delineation of new disorders, development of specific therapies, and better medical management for patients. Further rapid improvements will continue, improving the diagnostic utility of NGS. The medical genomics community should partner with patients to increase NGS access and equity and to drive therapeutics by using approaches such as drug repurposing, or engage the pharmaceutical industry with approaches that are scalable to larger groups of disorders to make it more feasible.

## Figures and Tables

**Table 1:** Case examples of diagnoses made by NGS, providing insights into pathogenicity

Gene	Insights into pathogenesis of rare disorder	Details of diagnosis	Details of pathogenesis
*MYBPC1* (MIM 160794)	Demonstrated a novel and expanded phenotype associated with this known gene (PMID 31264822)	Protein modeling and biochemical and kinetic studies demonstrated effect of variant on muscle structure/function	Novel phenotype for gene
*SETBP1* (MIM 611060)	Demonstrated a forme fruste presentation for a well-described and neurologically progressive disorder (PMID 32445275)	Documentation of a patient with a previously observed variant within a gene hotspot with a much milder phenotype/disease course than previously described	Novel phenotype–specific variant
*KMT2D* (MIM 602113)	Novel, and substantially different, phenotype associated with pathogenic variants within a 40-amino-acid region of the gene previously established to be associated with a different and well-described genetic syndrome (PMID 32083401)	Region of particular interest identified via variant sharing and review of data from both control databases and published cases	Novel phenotype–specific gene region
*IRF2BPL* (MIM 611720)	Novel disease gene reported in 2018, in which clinical phenotype significantly varies based on type of genetic change (PMID 30057031)	LOF variants cause severe neurodevelopmental regression, and missense variants cause a milder neurologic phenotype	Novel phenotype–specific type of variant
*SCN8A* (MIM 600702)	Individuals identified with variants resulting in protein LOF or GOF and different clinical presentations (PMID 25725044)	GOF variants cause severe early-infantile epileptic encephalopathy 13, while LOF variants cause intellectual disability with or without seizures	Novel phenotype–specific molecular consequence

Abbreviations: GOF, gain of function; LOF, loss of function; MIM, Mendelian Inheritance in Man; NGS, next-generation sequencing; PMID, PubMed reference number.
